# Supplementary material for: Disentangling metabolic functions of bacteria in the honey bee gut
Source: PLoS Biol. 2017 Dec 12;15(12):e2003467. doi: 10.1371/journal.pbio.2003467 (PMC5726620; doi:10.1371/journal.pbio.2003467)
Supplement: S8 Data — (ZIP) [file pbio.2003467.s008.zip › Z-score_plots/88 microbial substrate N-Cyclohexylformamide.pdf]

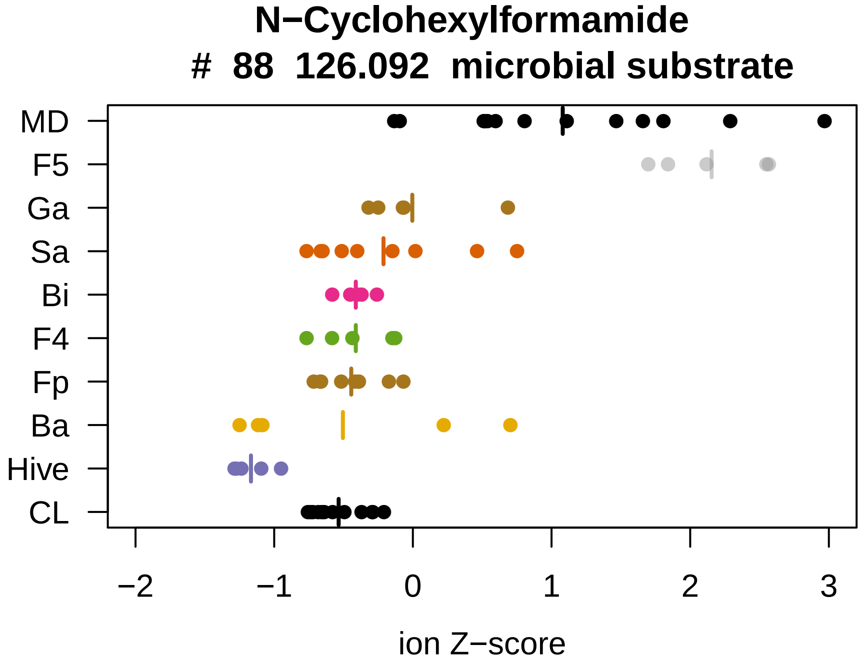
N−Cyclohexylformamide
88 126.092 microbial substrate
MD
F5
Ga
Sa
Bi
F4
Fp
Ba
Hive
CL
−2
−1
0
1
2
3
ion Z−score
